# Emergence of Hemagglutinin Mutations During the Course of Influenza Infection

**DOI:** 10.1038/srep16178

**Published:** 2015-11-05

**Authors:** Anna Cushing, Amanda Kamali, Mark Winters, Erik S. Hopmans, John M. Bell, Susan M. Grimes, Li C. Xia, Nancy R. Zhang, Ronald B. Moss, Mark Holodniy, Hanlee P. Ji

**Affiliations:** 1 Stanford Genome Technology Center, Stanford University, Palo Alto, CA, 94304, United States; 2 Division of Infectious Diseases and Geographic Medicine, Department of Medicine, Stanford University School of Medicine, Stanford, CA, 94305, United States; 3 VA Palo Alto Health Care System, Palo Alto, CA 94304, United States; 4 Division of Oncology, Department of Medicine, Stanford University School of Medicine, Stanford, CA, 94305, United States; 5 Depart of Statistics, The Wharton School, University of Pennsylvania, Philadelphia, PA 19104 USA; 6 Ansun BioPharma, Inc., San Diego, CA, 92121, United States

## Abstract

Influenza remains a significant cause of disease mortality. The ongoing threat of influenza infection is partly attributable to the emergence of new mutations in the influenza genome. Among the influenza viral gene products, the hemagglutinin **(HA)** glycoprotein plays a critical role in influenza pathogenesis, is the target for vaccines and accumulates new mutations that may alter the efficacy of immunization. To study the emergence of HA mutations during the course of infection, we employed a deep-targeted sequencing method. We used samples from 17 patients with active H1N1 or H3N2 influenza infections. These patients were not treated with antivirals. In addition, we had samples from five patients who were analyzed longitudinally. Thus, we determined the quantitative changes in the fractional representation of HA mutations during the course of infection. Across individuals in the study, a series of novel HA mutations directly altered the HA coding sequence were identified. Serial viral sampling revealed HA mutations that either were stable, expanded or were reduced in representation during the course of the infection. Overall, we demonstrated the emergence of unique mutations specific to an infected individual and temporal genetic variation during infection.

Influenza infection is a major cause of mortality worldwide^1^. Two influenza subtypes, A and B, cause the majority of infections. Influenza A’s viral genome has eight genes that encode 11 different proteins including hemagglutinin **(HA)** and neuraminidase **(NA)** glycoproteins. The HA glycoprotein targets the sialic acid residues on host respiratory epithelium and plays a critical role in influenza pathogenesis. Currently, there are 18 known HA types (i.e. H1, H2, etc.) with the majority of infections caused by H1 and H3. Within the HA protein are a number of residues which are antigenic sites - both H1 and H3 have at least five different epitopes that are know to be immunogenic^2^,^3^. It is these antigenic sites that serve as the targets for current influenza vaccine.

*De novo* mutations and other genetic variation is introduced into influenza’s genome via a number of mechanisms and they may have significant clinical consequences^4^. On a population level, antigenic drift is the result of new mutations that are introduced into currently circulating strains over time, while antigenic shift occurs when genetic segments are mixed with novel components, sometimes between species. Antigenic drift is attributable to influenza’s low fidelity RNA polymerase that lacks a function for error proof-reading. With each replication cycle, polymerase errors create *de novo* mutations, increasing the genetic diversity of the virus. As a result, mutations can occur in the HA gene^5^,^6^,^7^ and depending on their position, potentially alter HA’s antigenic epitopes.

Within a single infected individual, there are viral subpopulations or quasispecies characterized by novel mutations within various influenza genes such as HA^5^,^6^,^7^. We developed a robust and accurate next generation sequencing (NGS) approach to quantitatively detect new HA mutations and their representation in the overall viral population in an infected individual (Fig. 1)^8^. Using an Illumina sequencer, we readily generate ultradeep sequencing coverage greater than 10,000× from target genes (Flaherty *et al.*, Nucleic Acids Research, 40, 2012)^8^. To improve the sensitivity and specificity of detection of mutation even at very low allelic fractions that are representative of small quasispecies populations (e.g. less than 1.0%), we developed a Beta-Binomial statistical algorithm. This analysis method takes into account experimental variance from the molecular preparative assay (e.g. amplification, duplicated reads, etc.) and sequencing errors^8^. We minimize false positives in the variant calling and as a result, accurately determine the quantitative mutation allelic fraction (MAF) of a true mutation from experimental samples^8^. In a previous report, we used this methodology to accurately identify low abundance mutations in the NA gene among infected individuals.

For this study, we sought to characterize quantitatively mutations in the HA gene from a larger group of patients with H1N1 or H3N2 influenza infection. Our study had two goals. First, we sought to identify the presence of HA mutations that are present at a low fraction within the overall viral population present in an infected individual. These rare HA mutations are representative of new quasispecies within an infected individual and may have clinical implications for vaccines. Second, in a subset of these patients we determined the temporal variation in HA mutations using samples obtained at two separate time points from the same individual. During the course of an infection, temporal changes in these mutations representation provide insight about the possible origin of antigenic drift.

## Results

### Viral samples and their characteristics

A total of 17 subjects were involved in the study with confirmed H1N1 or H3N2 infection as determined with quantitative PCR specific to each subtype of the HA gene (Table 1). Subjects were otherwise healthy individuals. Seven patients had H1N1 infection and 10 patients had H3N2 infection. None of the individuals in this study received antiviral therapy and all of them recovered spontaneously. The age range was from 19 to 56 years with an average of 36.1 years. Nine subjects were men and eight were women.

The subjects presented with influenza-like illness (ILI) with an oral temperature of ≥ 100 °F ( ≥ 37.8 °C) and have one or more ILI symptom (cough, sore throat, nasal symptoms, headache, myalgia, sweat/chills, or prostration). Thus, samples listed as Day 1 represents the first sample taken from subjects in the study from which they were initially recruited (Table 1). Nasopharyngeal samples were obtained within 48 hours of symptom onset. H1N1 and H3N2 presence and viral loads (i.e. RNA copies/mL) were determined from the same quantitative HA PCR testing used for viral typing; sample viral load ranged from 7.2 × 10E5 to 1.18 × 10E8 copies/ml (Table 1).

Based on the viral load, we determined the viral copy number present in each sample. Those samples with low viral loads have a limited number of viral copies. With PCR amplification, starting with a low number of viral copies may lead to artifactual mutations. To reduce the possibility of spurious mutations as a result of PCR amplification error and bias, we used samples that had greater than 1.00 × 10E5 viral copies per amplification reaction. Per our calculations as detailed in the Methods, this viral copy number provides an adequate number of template molecules for detecting mutations at a MAF of 0.5% with high sensitivity and specificity. In addition, a subset of patients had viral samples collected longitudinally after the initial diagnosis (Table 1). This follow up sample was collected on either Day 3 or 5 during the course of active infection from one H1N1 patient and four H3N2 patients. They had adequate viral copy number for additional sequencing analysis.

### Overview of deep sequencing of the H1N1 and H3N2 HA gene

**Figure 1:**
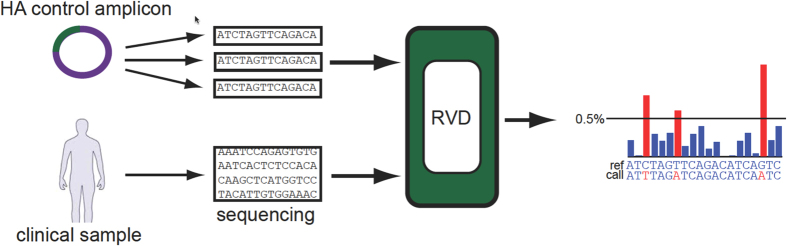
HA mutation analysis approach. Clinical HA samples were sequenced alongside three technical replicates of a control sample with known sequence. Sequence alignment was conducted. The rare variant detection method, RVD, was applied to sequencing output BAM files to call mutations using a minimum threshold of 0.5% mutation allele frequency (MAF).

Our sequencing analysis of the HA gene included a series of control replicates that were run in parallel with the experimental samples (Fig. 1). We used either a H1N1 or H3N2 HA gene amplicon that was subcloned into a plasmid; this was the starting template for the control replicates. The plasmid was colony purified to insure a clonal version was used in all experiments. Run in triplicate, this control enabled us to determine the extent of artifact mutations introduced from sequencing library preparation.

For sequencing, we relied on a PCR primer pair that produces a 1.6 Kb amplicon spanning the entire HA gene. We used a random insert transposase method for generation of the sequencing libraries. Direct amplicon sequencing was not practical because the size of the HA gene; this would require multiple PCR pairs which would significantly complicate the sequencing process and analysis. HA sequencing libraries were run on an Illumina MiSeq with 150 base paired-end reads. For alignment, we used the program BWA with HA references specific to one or the other subtype.

### High sensitivity HA mutation calling

The samples underwent sequencing on an Illumina MiSeq with 150 × 150 paired end reads. All the samples exceeded 10,000× average coverage with one exception; this sample had 9,077× average coverage. On the aligned sequence data, we used the RVD program^9^ for quantitative detection of mutations at low allelic fraction. As noted previously RVD relies on the sequence data from the control replicate to characterize a position specific error distribution. It models the error that may occur from different steps of the sequencing process (e.g. PCR amplification, library preparation, sequencing errors)^8^. Processing the control amplicon replicates and sample sequencing data simultaneously, RVD estimates the context-specific error at each base position for any given target gene^8^. This includes the wildtype “reference” reads versus the variant-containing “non-reference” reads and fits the model to the control replicate data. For example, setting the threshold p-value at *α* = 1 × 10^−6^ is equivalent to a Bonferroni corrected level of *α* = 1 × 10^−3^ for a 1 Kb target region and thus has a low false positive rate after controlling for multiple hypothesis testing.

With RVD, we determined the sensitivity and specificity of deep sequencing in detecting low abundance mutations. From our previous study (Flaherty *et al.*, 2012), we generated a receiver operator curve **(ROC)**. We demonstrated that with an average sequencing coverage of 10,000×, one has excellent power at a significantly low false positive rate to discriminate a variant fraction of 0.5%^8^. Even higher sensitivity at a MAF threshold of 0.1% is achievable although our previous studies have demonstrated a drop in specificity. Therefore, we relied on a 0.5% MAF threshold that provides a high specificity greater than 95%.

### Reproducibility of HA mutation detection

**Figure 2:**
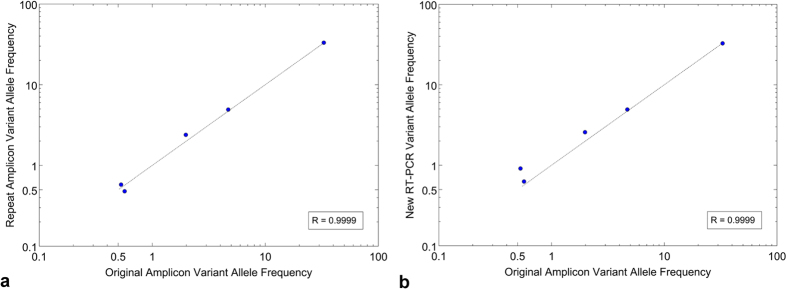
Mutation reproducibility demonstrated in independent experimental replicates. The reproducibility of quantitative mutation detection was determined with a series of independent experimental replicates. The effects of (**a**) PCR amplification and (**b**) reverse transcription were evaluated. An inclusive 0.5% fractional cutoff was used and all mutations found in one of the sample replicates were considered simultaneously. The correlation coefficient for each comparison was calculated and the best-fit slope was plotted.

To determine the reproducibility of quantitative mutation calling, we studied the effect of different steps in the sequencing library preparation. First, we examined the effect of PCR amplification on the sensitive detection of variants above the 0.5% threshold. For this experiment, we generated independent replicates with amplified HA products from the same viral sample (e.g. Patient 328011 with H1N1 infection). After library preparation, the independent replicates were sequenced and aligned to the HA reference. We achieved an average sequencing coverage of 34,000× on both samples and the sequence data was analyzed with RVD. Both replicates had a total of five common mutations at a MAF of 0.5% or higher. We compared the MAF values for each mutation as shown on a scatterplot (Fig. 2a) and demonstrated a correlation coefficient of greater than 0.999. Around the threshold of 0.5%, two mutations were found above 0.5% in one sample but slightly below 0.5% (0.47%) in the other.

We also determined the contribution of reverse transcription on mutation calling reproducibility. Using the same H1N1 viral RNA from patient 328011, we prepared independent experimental replicates starting with the reverse transcription step followed by amplification of the HA gene. Post-sequencing, we achieved an average sequencing coverage of 34,000× for two independent replicates. Like the PCR replicates, we identified the same five mutations between the two reverse transcription replicates. Figure 2b shows the mutation MAFs from the replicates in a scatter plot and again we demonstrated a high correlation coefficient of greater than 0.999. Overall, this analysis indicates the reproducibility of our mutation detection and MAF quantitative measurements.

### Deep sequencing the HA genes from individuals with H1N1 or H3N2 infection

We determined the number of viral copies required for accurate detection of mutations, using our MAF detection threshold of 0.5% (Methods). For example, if the library preparation uses a sample with 1,000 viral templates, accurate detection of a mutation with MAF of 0.1% would require amplification of a single mutant allele molecule. Overall, low viral copies per PCR amplification increases the likelihood of amplification bias and introducing PCR related artifacts. Based on our calculations using the viral loads of the samples, when one uses 1.0 × 10E5 viral templates or greater for each PCR amplification of the HA gene there is less chance of introducing of spurious mutations.

For any given sequencing run, three HA control replicates were run for either the H1N1 or H3N2 subtypes in parallel with the clinical samples. Data was generated using four separate sequencing runs. The sequence data was aligned to the HA reference specific to each subtype (See Methods). For all samples, the sequencing metrics are provided in Supplementary Table 1. Sequence data quality was high with 88% to 99% of the total number of sequence reads had base quality scores greater than 30. Alignment to the HA gene was greater than 88% of the reads and the average number of aligning reads for the H1N1 samples was 576,203 and for the H3N2 samples was 492,590. For the H1N1 samples, the average sequencing coverage ranged from 27,343× to 61,301×. For the H3N2 samples, the average sequencing coverage distribution ranged from a low of 9,077× to a high of 73,276×. Overall, the individual viral samples yielded high quality sequence data. All of the sequence data is available at the NIH Short Read Archive (SRA) with the accession identifier SRP051062.

### HA mutations and MAF quantitation from deep sequencing of H1N1 and H3N2 infections

Post-alignment, we conducted RVD analysis and identified HA mutations with a MAF greater than 0.5% (Supplementary Tables 2 and 3). A summary of the H1N1 and H3N2 HA mutation characteristics are presented in Table 2. From a total of 17 patients, 15 individuals had a HA mutation detected at a 0.5% MAF or greater in their influenza sample.

From the 22 influenza samples, we identified a total of 40 mutations of which 38 were unique. There were 35 unique SNVs and 3 deletions with sizes ranging from 1 to 5 bases. The quantitative allelic fraction (e.g. MAF) of the H1N1 and H3N2 HA mutations ranged from 0.50 to 66.00%. Among the H1N1 and H3N2 viral samples, all of the HA mutations were exclusive to a given patient.

**Figure 3:**
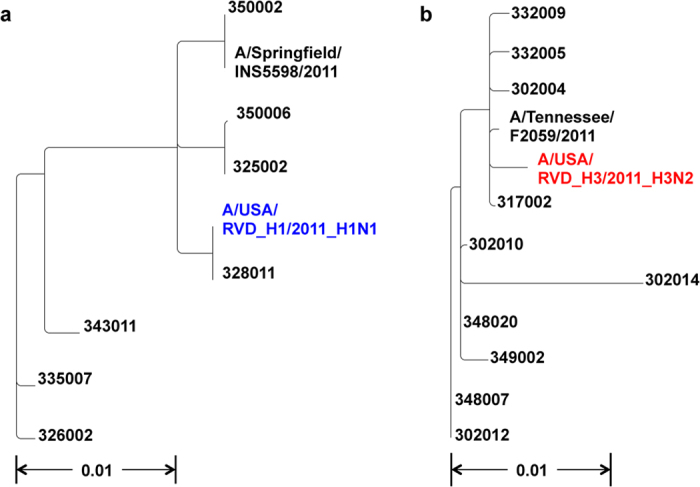
Phylogenetic trees comparing H1N1 and H3N2 clinical samples to known sequences. Phylogenies demonstrate the variation in sequence across the (**a**) H1N1 and **(b)** H3N2 clinical sample compared to the amplicon HA control sample sequences (A/USA/RVD_H1/2011_H1N1 and A/USA/RVD_H3/2011_H3N2) and the closest independent sequence isolate by BLAST search (A/Springfield/INS5598/2011 and A/Tennessee/F2059/2011). The scale bar indicates number of substitutions per nucleotide. Phylogenies were created using phylogenetic tree tools available at fludb.org.

As noted in the Methods, we determined the consensus sequence of the HA gene for each sample using the information available at the Influenza Research Database (http://www.fludb.org). Afterwards, we used PhyML^10^,^11^ to generate phylogenetic trees across the clinical samples (Fig. 3). The H1N1 samples were closest to the A/Springfield/INS5598/2011 isolate (Genbank CY129862.1). The H3N2 samples were closest to the A/Tennessee/F2059/2011 isolate (Genbank CY167636.1).

The only example of duplicated mutations occurred in longitudinal samples originating from the same individual (Patients 302014 and 317002) but at different time points. Overall, each mutation was unique to the viral population for any given patient. In addition, these results indicate that there was no inter-sample contamination or carryover that would lead to common mutations appearing.

We evaluated the fractional representation of the mutations at the time of Day 1. For H1N1 HA mutations, 68% (16/17) of the mutations had a MAF of less than 2%. Similarly for the H3N2 HA mutations, 70.5% (12/17) of the mutations had a MAF of less than 2%. These results suggest the emergence of *de novo* mutations, indicated by very low MAFs, during infection.

Several patients with H1N1 or H3N2 infection had mutations at a MAF greater than 10%. Three H1N1 patients (328011, 335007 and 35002) had mutations present that ranged from 19.60% to 32.90%. Four H3N2 patients had either nonsynonymous, synonymous or deletion mutations ranging from 11.70% to 61.10%. Infections that have mutations with higher MAF values may be indicative of mixed infections where the individual host has been infected with multiple viral variants^6^.

**Figure 4:**
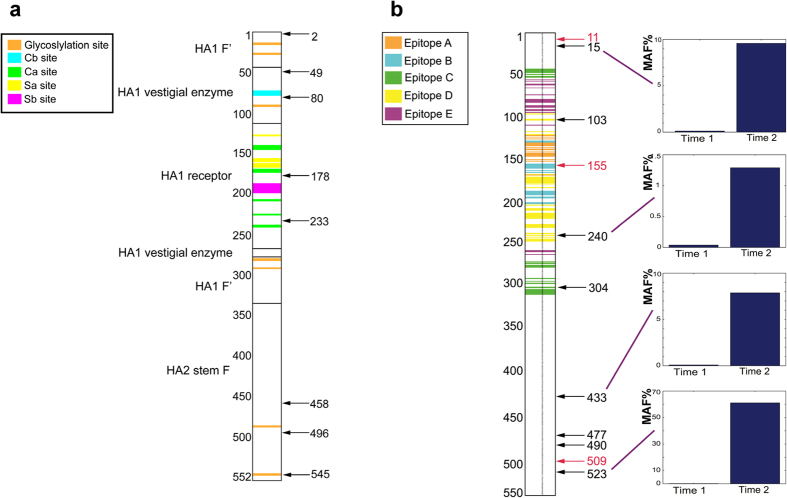
Domain map of the HA gene with location of coding mutations. (**a**) The coding region of the H1N1 HA gene is shown as a vertical bar with colored protein domains as noted in the legend. Amino acid number is listed on the left side. All of the coding mutations are shown as black arrows. Coding mutations were aligned to previously described antigenic and glycosylation sites (20602265, 20339031)^13^,^14^. S545F was found in a previously identified glycosylation site. (**b**) The overall coding regions of the H3N2 HA gene is shown as a vertical bar with colored protein domains as per the legend^15^. Amino acid number is listed on the left side. All of the coding mutations [black arrows] and deletions [red arrows] were aligned to previously described H3 epitopes^15^. On the left of the domain and mutation map, the expanded coding mutations from Patient 328011 are shown on a linear MAF histogram plot. Of the eight mutations, P103S and G240R occurred in Epitope D and A304T occurred in Epitope C. One of the three deletions (C155) occurred in Epitope B.

Among the 38 missense mutations, 19 altered the amino acid composition of the HA gene (Table 2; Fig. 4). For H1N1, there were eight nonsynonymous mutations leading to amino acid substitutions. For H3N2, there were 11 nonsynonymous mutations leading to amino acid substitutions. To determine the potential functional consequences of the amino acid substitutions, we used the program PROVEAN^12^. This program determines the degree of amino acid conservation compared to other sequences and provides a score for the impact of the variant on protein function. It has been used for interpreting the impact of amino acid substitutions in influenza genes. A score of less than −2.5 indicates a high probability of deleterious changes to the protein’s function. We also considered the amino acid position of the mutation compared to HA domains that have been previously characterized.

For the H1N1 results, three missense mutations were predicted to have a significant impact on HA gene function (Table 2; Supplementary Table 2). As shown in Fig. 4a, amino acid substitutions occurred within proximity of important functional domains of HA. Interestingly, two of these mutations may have an impact on antigenicity. Patient 328011 had a S545F residue alteration that occurred in a glycosylation site^13^; this mutation may have a significant impact on HA antigenicity^13^. Patient 35002 had a S80P substitution that is adjacent to the Cb site that is within the vestigial esterase domain structurally determined by Xu *et al.*^14^. This mutation’s PROVEAN score (−3.149) suggests a major impact on HA function.

Based on the analysis of the H3N2 results, five missense mutations were predicted to have a functional impact (Table 3; Supplementary Table 3). Patient 302010 had a G240R substitution that directly alters a H3N2 epitope^15^. This mutation may have a possible effect on antigenicity. There were a number of mutations that affected epitopes but did not have a significant Provean score. For example, Patient 348020 had a mutation A304T substitution affecting Epitope C and Patient 317002 had a P103S substitution that alters Epitope D^15^. Determining the functional consequences of these mutations will require further study.

Several deletions were identified in the H3N2 HA gene (Table 3). Patient 317002 had a three bp deletion that was in frame and thus maintained the HA open reading frame. The functional consequences of in-frame mutations on HA gene function are unknown. For Patient 332005, a single base deletion occurred at HA nucleotide **(nt)** position 464. This leads to a frame shift in the HA gene and alters epitope B^15^. Patient 302004 had a five base deletion at HA nucleotide position 1527 and alters the frame past codon 509 which is proximal to the C-terminus.

To determine whether the mutations we identified had any overlap with antigenic drift from the 2104/2015 influenza season, we conducted a review of the literature based on a PubMed search conducted on 6/9/2015 with the following search terms: “2014 2015 influenza antigenic drift”; “H3N2 antigenic drift”; “H3N2 influenza A drift”; “2014 H3N2 drift”). We identified a study by Skowronski *et al.*, that examined the presence of antigenic drift from the 2014/2015 influenza season for the H3N2 subtype^16^. We did identify a HA frameshift mutation at position 155 that is localized to the H3 cluster transition region and may be a key residue for receptor binding. Skowronski *et al.* identified HA mutations at positions 156 and 159 from the 2014/2015 season. Otherwise, there were no overlapping mutations. Given that there is a three-year interval between this study and ours and the small number of patients in our study, the lack of common mutations is not surprising.

### Temporal changes in HA mutations during infection

One H1N1 and four H3N2 patients had longitudinal influenza samples with viral loads adequate for mutation detection at the 0.5% MAF threshold (Table 4). We analyzed the sequence data as previously described and determined the MAF of the mutation for both time points (i.e. Time 1 and Time 2). For any given mutation, we calculated the fold change (FC) of the MAF between the two separate days. We employed a Z-statistic (Methods) to determine if the FC per a mutation was significant. Our analysis included a Bonferroni correction based on the overall length of the HA gene (e.g. approximately 1.7 Kb). All of the MAF longitudinal changes were found to be statistically significant (P < 0.0002) with the exception of two mutations that showed no significant MAF changes between the two time points.

From these longitudinal samples, we identified a total of 16 mutations with a MAF of 0.5% or greater. Ten of these mutations lead to amino acid substitutions and the results are summarized in Table 4. The coding mutations leading to amino acid substitutions, deletions and frameshifts are mapped in the vicinity of H3N2 HA domains (Fig. 4b). The remaining 14 mutations were above the 0.5% threshold at only a single time point, and underwent a significant MAF reduction during the course of infection. Among the five non-coding mutations in this set, four were detected at the first time point and saw reduced MAF at time point 2. One mutation was only detected at the second time point.

From the single H1N1 patient (328011), five mutations were identified at Day 1 with a MAF greater than 0.5%. On Day 3, all of these mutations dropped below the 0.5% MAF level. This included a synonymous mutation at nucleotide position 1285; on Day 1 the MAF was 32.90% but subsequently dropped below 0.5%. The other mutations occurred at a MAF less than 5%, suggesting that there were different quasispecies present. The overall reduction in genetic diversity suggests that quasispecies were eliminated during the course of the 328011’s influenza infection and correlates with the drop in viral load that occurred between the two days.

For patient 30210, five HA mutations demonstrated an overall expansion between Days 1 and 3 (Table 4 and Fig. 4b). All of these mutations had a MAF of less than 0.5% at Day 1 but expanded to as high as 61.10%. This includes four mutations that lead to amino acid substitutions. Among the expansions was the G240R substitution that alters the H3N2 epitope D amino acid residue. Interestingly, the synonymous mutation demonstrated the smallest fold change compared to the mutations leading to amino acid substitutions. There was a slight increase in viral load between Days 1 and 3 (Table 1) where the log 10 viral loads went from 5.981 to 6.034. The increase in mutation MAF may represent random population drift or be the result of a selective advantage linked to antigenic changes. Interestingly, Patient 30210 had an increase in viral load between the two time points.

Patient 302012 had a single mutation (e.g. nt position 489 A- > T) that did not alter the amino acid residue. This mutation had a MAF of 0.85% and was practically eliminated between Days 1 and 3.

Patient 302014 had two mutations. The first mutation (nt position 129 T- > C) was synonymous, did not lead to an amino acid substitution and remained stable in MAF during the two time points. The second mutation (nt position 1469 T- > C) leads to V490A residue change and showed a reduction in MAF below the 0.5% threshold. These results suggest the presence of at least two distinct quasispecies populations.

Patient 317002 had an in-frame three bp deletion that had a stable MAF from Days 1 and 3 with no statistically significant change. The other two alterations included a nonsynonymous (P103S substitution) and a synonymous mutation (nt position 1458 T- > C). The second mutation had an initial MAF less than 1.0% that was further reduced below the 0.5% MAF threshold. These results suggest that the quasispecies populations can follow different trends in size during infection.

## Discussion

We quantitatively detected HA mutations at a low fractional composition of the overall viral population. Our study involved H1N1 and H3N2 influenza subtypes. This study demonstrates the presence and emergence of genetic diversity during active influenza infection. Recent studies such as Ghedin *et al.* showed that genetic diversity within individual influenza samples but few of these studies have examined mutation composition and longitudinal changes during active initial infection^6^. Overall, our study demonstrates the rapid emergence and fluctuations of novel mutations, an indicator of influenza quasispecies during active infection. Our results also suggest that antigenic drift is present relatively early during initial infection.

Among the eight coding point mutations that were predicted to be deleterious, the substitutions located adjacent to antigenic and glycosylation sites may alter the antigenic properties. Not surprisingly, these mutations had not been noted in a previous survey of the influenza antigenic drift from the 2014/2015 influenza season. Given that our small population was recruited from the 2011 season, the lack of overlap is not suprising.

To better define the role of novel HA mutations in antigenic drift will require additional characterization of these mutations from more samples. Future longitudinal studies of clinical populations will provide us with a better understanding of the functional significance of these novel mutations. Expanded deep sequencing of multiple influenza genes may help differentiate between background drift and drift related to host immune interactions. We are planning for such studies in the future. Overall, our results are supportive of the enormous potential for deep sequencing methods to provide additional insight about how the emergence of new influenza mutations impacts clinical outcomes.

## Methods

### H1N1 and H3N2 sample preparation

This study was conducted in compliance with the Helsinki Declaration and in accordance with approved experimental guidelines required by Stanford University. For all patients cited in this study, we obtained informed consent to conduct research. The study protocol and the experimental methodologies were approved by the Institutional Review Board (IRB) at Stanford University School of Medicine.

These individuals participated in a randomized, double blind, placebo-controlled phase 2B clinical trial and were in the placebo arm^17^. Individuals whose samples were analyzed in this study were enrolled in 8 different states (UT, CA, OK, FL, TX, OR, LA, CO, TN) across the United States^17^. It could not be confirmed whether any of the subjects could have been in contact with one another as a few subjects were enrolled in the same clinical centers but at different timeframes. The subjects were healthy individuals who presented with influenza-like illness (ILI) who later had laboratory confirmed influenza infection. Subjects were to be febrile with an oral temperature of ≥ 100 °F ( ≥ 37.8 °C) and have one or more ILI symptom (cough, sore throat, nasal symptoms, headache, myalgia, sweat/chills, or prostration). Samples were obtained at the time of presentation and this is listed as Day 1.

Nasopharyngeal samples were collected in viral transport media. Patients were confirmed to be the H1N1 or H3N2 influenza subtypes by quantitative PCR from these samples. The quantitative viral loads were determined (ViraCor-IBT, Lee’s Summit, MO)^17^. Viral load measurements (RNA copies/mL) were used to calculate the number of copies of influenza RNA per PCR amplification. The PCR target for each of the different subtypes of influenza A viruses was gene segment 4 (encodes for the hemagglutinin protein, HA). Our calculation considers the samples and the various aliquot volumes used for each step.

### Consideration of viral copies for detecting mutations with sequencing

We identified samples for which the total number of viral copies was sufficient to accurately identify mutations at lower allelic fractions. Viral copies are derived from the viral load as measured with quantitative PCR. Thus, we can determine the number of template viral molecules that are used steps of the viral RNA and sequencing library preparation.

We determined a threshold for viral copies for our analysis. The MAF derived from sequencing coverage data can be modeled as a binomial random variable with mean equal to (viral load)*(mutant allele frequency). Our estimate of the MAF is based on the proportion of reads at a site carrying the mutant allele (normalized to remove background sources of bias). The ratio of the standard error to mean of the mutant allele frequency estimate is approximately equivalent to 1/sqrt (viral copies per sequencing reaction * mutant allele fraction) for lower MAF values. For low viral copies used in a given HA gene PCR amplification, this ratio becomes increasingly large and points to the higher likelihood of spurious mutations introduced through amplification or sequencing. Thus, we restricted our analysis to those viral samples for which this ratio is larger than q for variants with mutation allele frequency less than r. The lower threshold on n should be 1/(rq^2). To achieve a value where r = 0.005 (MAF detection of 0.5%) and a q = 0.4, the threshold would be 100,000 viral copies per a PCR amplification.

### Sequencing library preparation

From the viral media for the nasal swab, we used 140 μl for RNA processing. Influenza RNA was extracted using Qiagen Viral RNA Mini Kits (Qiagen, Valencia, CA) in a total volume of 50 μl eluent. From this RNA, we used 10 μl of RNA for Superscript II RT reverse transcription with the primer Uni12 (Hoffmann, 2001) in a total volume of 20 μl (Life Technologies, Grand Island, NY). Afterwards, 5 μl of the cDNA was amplified using the Expand High Fidelity PCR System (Roche Applied Sciences, Indianapolis, IN) and primer HANS-890R (Hoffmann) and primer HA-H3mw (AGCAAAAGCAGGGGATAATTC) for H3 samples, or primer HA-H1mw (AGCAAAAGCAGGGGAAAAC) for H1 samples. Cycling parameters were 1 cycle of 94 °C for 30 sec, 40 cycles of 94 °C for 15 sec, 58 °C for 15 sec, 72 °C for 5 min, and 1 cycle of 72 °C for 10 min. The PCR products were purified using Qiagen PCR purification kits. Follow-up sample time points were chosen based on whether there was a sufficient amount of viral RNA for analysis.

Sequencing libraries were prepared from the H1 and H3 amplicons with the Nextera XT Sample Prep kit and Nextera XT 24 index kit (Illumina, San Diego, CA). A total of 3 ng of amplicon was incubated with the Nextera transposon enzyme for 1 minute at 55 °C. Subsequently, the sequencing library was amplified for 12 cycles, introducing multi-sample indexes and sequencing adapters as per the manufacturer’s protocol (Illumina). PCR parameters were 1 cycle of 72 °C for 3 minutes, 1 cycle of 95 °C for 30 seconds, 12 cycles of 95 °C for 10 seconds, 55 °C for 30 seconds, 72 °C for 30 seconds, and 1 cycle of 72 °C for 5 minutes after which samples were held at 10 °C. The PCR reaction was purified using 30 μl (0.6 × volume) AMPure XP beads (Beckman Coulter) and eluted in 15 μl elution buffer. Size profiles of libraries were analyzed with the Bioanalyzer High Sensitivity DNA assay (Agilent, Santa Clara, CA). The concentration of the sequencing libraries was determined by Qubit dsDNA HS assay (Invitrogen, Carlsbad, CA).

As described by Flaherty *et al.*, our detection algorithm relies on high coverage sequence data generated from control gene amplicons in replicates^8^,^9^. We generated an independent set of HA control amplicons originating from a single, clonally derived plasmid with the HA gene. Sequencing the replicate HA amplicons enables us to determine the total experimental variance introduced by PCR amplification, the DNA sequencing library preparation and next generation sequencing with an Illumina system. The control amplicon sequencing occurs in the same sequencing run as the clinical samples.

To construct this plasmid, selected PCR products derived from influenza viral samples were cloned using TOPO TA cloning kits (Life Technologies, Grand Island, NY) per the manufacturer’s instructions and transformed into E. coli. We isolated individual clones of the transformed E. coli to insure clonal copy of the vector. Afterwards, an individual plasmid was subject to Sanger sequencing to confirm the HA gene sequence. With clonally-isolated plasmid as template, we used the same primers, PCR conditions and sequencing library method to generate at least three control replicate amplicons as the influenza clinical samples. As a final step, sample specific indexes were added to each of three libraries. Sequencing libraries were quantitated on both an Agilent Bioanalyzer and a fluorescent assay.

### Deep sequencing of the HA gene, alignment and mutation analysis

The indexed sequencing libraries were pooled together in equal concentrations. This included libraries from the clinical sample HA gene and the control amplicon replicates that were run in triplicate to generate the error distribution necessary for variant calling. The indexed libraries were sequenced at a concentration of approximately 10 pM on an Ilumina MiSeq with 150 by 150 paired-end reads using a V1 reagent cartridge (Illumina). The Illumina MiSeq software (MCS v2.0.5) performed read trimming to remove adapter sequence and binning of indexes to separate fastq files. Indexing assignment for sequence reads to the appropriate sample relied on the standard Illumina software.

All samples were aligned using the program BWA 0.6.2 with the default single-end sequence alignment parameters. For our references, we aligned the H1 samples, to the reference sequence RVDH1/2011_H1N1 (Genbank KF848937.1) and the H3 samples were aligned to the RVD_H3/2011_H3N2 reference sequence (Genbank KF848938.1). Both reference sequences were approximately 1.7 Kb in length. The reference HA coordinates relied on the sequences provided by Soudararajan *et al.* for H1^2^ and Wilson *et al.* for H3^18^. The protein sequences of H1 and H3 controls were determined from the nucleotide sequences using fludb.org sequence annotation tool (http://www.fludb.org). The sequence data is available at the Short Read Archive (SRA) with the accession identifier SRP051062.

### Analysis of HA mutations

After alignment, analysis for single nucleotide variants **(SNVs)** was conducted with the program RVD (http://dna-discovery.stanford.edu/software/rvd/)^9^. For the program’s parameters, we used sequence data with a base quality score of 30 or greater. We chose a minimum threshold of 0.5% MAF to call mutations which we previously demonstrated provides greater than 95% sensitivity and specificity when one achieves an average 2,000× sequencing coverage^9^. All mutations were visually confirmed with program IGV^19^. We only report mutations that were not found in the sequence data from the control amplicon replicates. After variant calling, we identified the nonsynonymous mutations and determined the potential consequences of amino acid substitutions with the program PROVEAN using the default setting^12^. In the case of our analysis we used the default cutoff of −2.5 to classify a neutral versus deleterious mutations.

We determined the majority consensus HA sequence. After RVD analysis, we identified the positions in the HA gene with a homozygous allele distinct from the reference sequence used for alignment. These homozygous positions were used to create a HA sequence consensus. We generated phylogenetic trees comparing the consensus nucleotide sequences of our clinical samples with those of the H1 and H3 reference sequences and the closest isolates by BLAST search as previously listed. For this analysis we used PhyML^10^,^11^ with default settings (http://www.fludb.org).

For calling insertion and deletions (indels), we relied on the program Varscan2^20^. For this analysis, we used the following settings: a minimum total sequencing coverage of 8 reads; a minimum number of variants seen in at least 2 non-duplicated reads; representation on both forward and reverse reads; a p-value of 0.05. The control amplicons were included in the indel analysis as well. Afterward, we validated the indel variants in the clinical samples with IGV and eliminated all indels that were also present in the three control amplicons.

### Longitudinal comparison during viral infection

Five patients had two samples taken at two different time points with adequate number of viral copies. These samples were used to determine longitudinal differences in the fractional representation of mutations for a specific patient. First, we identified the HA mutations with a 0.5% MAF or greater and compared it to the other time point. Given that many of the mutations from the secondary time point had a MAF lower than the 0.5% threshold, this required determining the actual read coverage of the mutation versus the read coverage for the reference base. As a secondary check, comparison to the amplicon controls data was also used to eliminate false positives.

To determine the statistical significance of MAF differences from two time points, we used the test of proportions (e.g. Z-test). For a given position, we considered the overall number of reads for a position and proportion of variant reads in Time 1 versus Time 2. We determined the read depth of the variant and wildtype allele for the mutation in question from each time point. The PROC FREQ procedure in SAS 9.2 (SAS Institute Inc, Cary NC) was used for this analysis and generated a p value fore each Z-score. Our significance threshold was P < 0.0000294 after the Bonferroni correction based on the overall bp length of the HA gene (1.6 Kb).

## Additional Information

**How to cite this article**: Cushing, A. *et al.* Emergence of Hemagglutinin Mutations During the Course of Influenza Infection. *Sci. Rep.* **5**, 16178; doi: 10.1038/srep16178 (2015).

## Supplementary Material

Supplementary Tables

## Figures and Tables

**Table 1:** Influenza sample characteristics.

Influenza Subtype	Patient Identifier	Age (years)	Gender	Day of Sample*	Viral Loadǂ (copies/ml)	Viral Load Log 10 (copies/ml)
H1N1	325002	31	F	1	6.056E + 06	6.782
	326002	23	F	1	1.653E + 06	6.218
	328011	42	M	1	5.535E + 06	6.743
				3	7.199E + 05	5.857
	335007	19	M	1	2.761E + 06	6.441
	343011	56	F	1	1.181E + 07	7.072
	350002	38	M	1	3.463E + 06	6.539
	350006	30	F	1	1.712E + 06	6.234
H3N2	302004	42	M	1	1.257E + 07	7.099
	302010	32	M	1	9.561E + 05	5.981
				3	1.081E + 06	6.034
	302012	32	M	1	1.114E + 08	8.047
				3	3.370E + 06	6.528
	302014	23	M	1	3.754E + 06	6.574
				5	1.705E + 06	6.232
	317002	37	F	1	1.184E + 08	8.073
				3	2.217E + 07	7.346
	332005	39	F	1	4.703E + 07	7.672
	332009	55	M	1	2.484E + 07	7.395
	348007	47	F	1	3.605E + 06	6.557
	348020	44	F	1	7.238E + 06	6.860
	349002	23	M	1	4.213E + 06	6.625

^*^Day 1 is defined as the first day a sample was collected and was required to be collected within 48 hours of symptom onset. ǂThe influenza hemagglutinin gene was used as a PCR target to determine influenza viral load copy number.

**Table 2:** Summary of HA mutations.

Influenza Subtype	H1N1	H3N2
Total Number of Unique HA Mutations	16	22
HA Nonsynonymous Mutations	8	11
Predicted Deleterious HA Mutations	3	5
Average HA Mutations Per Sample	2.0	1.6
Total Number of Viral Samples	8	14
Viral Samples with HA Mutations > 0.5% MAF	7	10
Viral Samples with Nonsynonymous Mutations	6	7

**Table 3:** HA mutations leading to amino acid substitutions, deletions or frame shifts.

Subtype	Patient ID	Day of Sample*	HA Gene Nt Position	Reference Nt	Variant Nt(s)	Mutation Allelic Fraction (%)	HA Amino Acid Position	Reference Amino Acid	Amino Acid Change	Domain	Provean score
**H1N1**	325002	1	146	G	A	1.35	49	G	E		−4.208
	326002	1	532	G	A	0.64	178	V	M	adjacent to Ca site	−1.118
	328011	1	697	T	C	4.70	233	Y	H	adjacent to Ca site	−2.431
			1372	A	G	0.57	458	N	D		1.423
			1634	C	T	0.58	545	S	F	glycosylation site	−3.184
	335007	1	6	T	G	21.63	2	N	K		0.598
	343011	1	1487	C	T	0.65	496	A	V		−2.353
	350002	1	238	T	C	1.84	80	S	P	adjacent to Cb site	−3.149
**H3N2**	302004	1	1430	G	A	0.72	477	C	Y		−7.835
			1527	AGGGT	-AGGGT	0.52	509	S	frameshift		
	302010	3	44	T	C	9.57	15	L	P		−4.512
			718	G	A	1.29	240	G	R	Epitope D	−4.130
			1297	A	G	7.87	433	N	D		−3.566
			1567	T	C	61.10	523	S	P		−2.903
	302014	1	1469	T	C	2.30	490	V	A		−0.402
		1	31	GCA	-GCA	1.31	11	A	in frame		
	317002		307	C	T	0.86	103	P	S	Epitope D	−0.805
		3	31	GCA	-GCA	1.28	11	A	in frame		
	332005	1	464	C	-C	10.40	155	T	frameshift	Epitope B	
	348020	1	910	G	A	0.69	304	A	T	Epitope C	−0.386

^*^Day 1 is defined as the first day a sample was collected and was required to be collected within 48 hours of symptom onset.

**Table 4:** Longitudinal analysis of HA mutations.

Subtype	Patient ID	HA Gene Nt Position	Reference Nt(s)	Variant Nt(s)	Time 1 Mutation Allelic Fraction (%)	Time 2 Mutation Allelic Fraction (%)	Fold Change (Time 1 / Time 2)	HA Amino Acid Position	Reference Amino Acid	Amino Acid Change
H1N1	328011	519	A	G	1.98	0.02	0.010	173	G	None
		697	T	C	4.70	0.01	0.002	233	Y	H
		1285	T	C	32.92	0.15	0.004	429	L	None
		1372	A	G	0.57	0.01	0.021	458	N	D
		1634	C	T	0.53	0.00	0.000	545	S	F
H3N2	302010	44	T	C	0.12	9.57	81.085	15	L	P
		718	G	A	0.04	1.29	33.947	240	G	R
		1297	A	G	0.09	7.87	91.558	433	N	D
		1567	T	C	0.11	61.10	535.956	523	S	P
		1591	T	C	0.05	1.23	27.289	531	L	None
	302012	489	A	T	0.85	0.02	0.021	163	A	None
	302014	129	T	C	4.09	6.77	1.654	43	V	None
		1469	T	C	2.30	0.36	0.157	490	V	A
	317002	31	GCA	-GCA	1.31	1.28	0.979	11	A	Codon deletion
		307	C	T	0.86	0.18	0.206	103	P	S
		1458	T	C	0.77	0.14	0.176	486	Y	None

Nt = nucleotide
