# Supplementary material for: Emergence of Hemagglutinin Mutations During the Course of Influenza Infection
Source: Sci Rep. 2015 Nov 5;5:16178. doi: 10.1038/srep16178 (PMC4633648; doi:10.1038/srep16178)
Supplement: Supplementary Tables [file srep16178-s1.pdf]

SUPPLEMENTARY MATERIALS

Title: Emergence of Hemagglutinin Mutations During the Course of Influenza Infection

Anna Cushing[1], Amanda Kamali[2], Mark Winters[3], Erik S. Hopmans[1], John M. Bell[1], Susan M. Grimes[1], Li C. Xia[4], Nancy R. Zhang[5], Ronald B. Moss[6], Mark Holodniy[2,3], Hanlee P. Ji[1,4,*]

[1]Stanford Genome Technology Center, Stanford University, Palo Alto, CA, 94304, United States

[2]Division of Infectious Diseases and Geographic Medicine, Department of Medicine, Stanford University School of Medicine, Stanford, CA, 94305, United States

[3]VA Palo Alto Health Care System, Palo Alto, CA 94304, United States

[4]Division of Oncology, Department of Medicine, Stanford University School of Medicine, Stanford, CA, 94305, United States

[5]Depart of Statistics, The Wharton School, University of Pennsylvania, Philadelphia, PA 19104 USA

[6]Ansun BioPharma, Inc., San Diego, CA, 92121, United States

* To whom correspondence should be addressed.

Hanlee P. Ji, MD

Division of Oncology, Department of Medicine – Stanford University School of Medicine

CCSR 1115, 269 Campus Drive

Stanford, CA 94305-5151

Email: genomics_ji@stanford.edu

Phone: 650-721-150

Supplementary Table 1. Metrics of HA gene sequencing.

Influenza Subtype	Patient Identifier	Day of Sampling	Total Sequence Reads	Mapped Sequence Reads	Percentage Mapped Reads	Average HA Gene Coverage
	325002	1	423,096	393,307	92.96%	27,343
	326002	1	651,304	585,673	89.92%	44,281
	328011	1	530,900	495,616	93.35%	40,031
H1N1	328011	3	487,694	439,488	90.10%	35,283
	335007	1	978,172	876,080	89.56%	59,372
	343011	1	993,650	906,743	91.25%	61,301
	350002	1	814,590	757,509	92.99%	51,647
	350006	1	799,136	737,761	92.32%	49,767
	302004	1	111,146	103,762	93.36%	9,077
	302010	1	169,738	159,274	93.84%	14,084
	302010	3	190,474	173,872	91.28%	13,375
	302012	1	529,354	507,339	95.84%	39,997
	302012	3	699,512	669,952	95.77%	52,822
	302014	1	600,948	536,229	89.23%	44,561
H3N2	302014	5	451,286	402,520	89.19%	33,382
	317002	1	716,530	673,322	93.97%	53,742
	317002	3	692,436	653,761	94.41%	42,141
	332005	1	750,444	711,377	94.79%	57,011
	332009	1	721,122	685,217	95.02%	55,089
	348007	1	568,212	537,483	94.59%	43,239
	348020	1	573,478	543,099	94.70%	43,801
	349002	1	996,344	927,651	93.11%	73,276

Supplementary Table 2. HA mutations in H1N1.

Influenza Subtype	Patient Identifier	Day of Sample	Viral Load	Log Viral Load	Viral Copies Per Analysis	HA Gene Nt Position	Reference Nt	Variant Nt(s)	Mutation Allelic Fraction (%)	HA Amino Acid Position	Reference Amino Acid	Amino Acid Substitution	Provean Score
**H1N1**	325002	1	6.056E+06	6.782	4.239E+05	146	G	A	1.35	49	G	E	-4.208
	326002	1	1.653E+06	6.218	1.157E+05	532	G	A	0.64	178	V	M	-1.118
						1102	C	T	1.49	368	L	L	
	328011	1	5.535E+06	6.743	3.874E+05	519	A	G	1.98	173	G	G	
						697	T	C	4.70	233	Y	H	-2.431
						1285	T	C	32.92	429	L	L	
						1372	A	G	0.57	458	N	D	1.423
						1634	C	T	0.58	545	S	F	-3.184
	335007	1	2.761E+06	6.441	1.933E+05	6	T	G	21.63	2	N	K	0.598
	343011	1	1.181E+07	7.072	8.266E+05	306	G	A	1.00	102	E	E	
						1487	C	T	0.65	496	A	V	-2.353
	350002	1	3.463E+06	6.539	2.424E+05	238	T	C	1.84	80	S	P	-3.149
						706	C	T	19.55	236	L	L	
						1206	C	T	28.12	402	H	H	
						1536	G	A	1.86	512	R	R	
	350006	1	1.712E+06	6.234	1.199E+05	1209	G	A	0.56	403	L	L	

Supplementary Table 3. HA mutations in H3N2.

Influenza Subtype	Patient Identifier	Day of Sample	Viral Load	Log Viral Load	Viral Copies Per Analysis	HA Gene Nt Position	Reference Nt	Variant Nt(s)	Mutation Allelic Fraction (%)	HA Amino Acid Position	Reference Amino Acid	Amino Acid Substitution	Provean Score
H3N2	302004	1	1.257E+07	7.099	8.800E+05	204	T	C	0.51	68	D	D	
						636	T	A	1.03	212	A	A	
						1430	G	A	0.72	477	C	Y	-7.835
						1527	AGGGT	-AGGGT	0.52	509	S	frameshift	
						1548	A	C	11.70	516	L	L	
	302010	3	1.081E+06	6.034	7.565E+04	44	T	C	9.57	15	L	P	-4.512
						718	G	A	1.29	240	G	R	-4.13
						1297	A	G	7.87	433	N	D	-3.566
						1567	T	C	61.10	523	S	P	-2.903
						1591	T	C	1.23	531	L	L	
	302012	1	1.114E+08	8.047	7.801E+06	489	A	T	0.85	163	A	A	
	302014	1	3.754E+06	6.574	2.627E+05	129	T	C	4.09	43	V	V	
						1469	T	C	2.30	490	V	A	-0.402
		5	1.705E+06	6.232	1.193E+05	129	T	C	6.77	43	V	V	
	317002	1	1.184E+08	8.073	8.285E+06	31	GCA	-GCA	1.31	11	A	in frame	
						307	C	T	0.86	103	P	S	-0.805
						1458	T	C	0.77	486	Y	Y	
		3	2.217E+07	7.346	1.552E+06	31	GCA	-GCA	1.28	11	A	in frame	
	332005	1	4.703E+07	7.672	3.292E+06	464	C	-C	10.40	155	T	frameshift	
	348007	1	3.605E+06	6.557	2.524E+05	240	A	G	0.74	80	Q	Q	
	348020	1	7.238E+06	6.860	5.067E+05	495	C	T	1.05	165	N	N	
						910	G	A	0.69	304	A	T	-0.386
						1293	G	A	65.64	431	L	L	
						1521	G	A	0.992	507	L	L	
